# Adolescent Psychosis and Rectal Prolapse

**DOI:** 10.7759/cureus.27615

**Published:** 2022-08-02

**Authors:** Autumn D Pak, Tien T Nguyen, Mathew Bogoyas

**Affiliations:** 1 Psychiatry, Washington State University, Richland, USA; 2 Psychiatry, Elson S. Floyd College of Medicine, Spokane, USA

**Keywords:** antipsychotic medications, constipation, adolescent, psychosis, rectal prolapse

## Abstract

Psychosis is a constellation of symptoms that present with a disconnect from reality. The duration, severity, and presentation of symptoms can present on a wide spectrum, and etiologies can vary from patient to patient. Psychosis is also associated with self-injurious thinking, behavior, and suicidality. Long-term treatment of psychosis with antipsychotics can often result in side effects like constipation, sedation, dry mouth, and metabolic syndrome. Though rectal prolapse is uncommon in adolescent patients, there was a noted correlation with rectal prolapse in adult patients that were treated for chronic psychiatric disease. We report a case of a 17-year-old female with psychosis and rectal prolapse, who was admitted for inpatient treatment.

## Introduction

Psychosis is a loss of touch with reality that can present with a variety of clinical symptoms [1]. For the context of this paper, the symptoms and conditions of psychosis exist along a spectrum and are only a part of the diagnosis of psychotic disorders. An estimated 1.5-3.5% of people will meet diagnostic criteria for a psychotic disorder, though much higher numbers will present with at least one symptom of psychosis in their lifetime. The peak onset of an episode of psychosis is the late twenties for females and teens to early twenties for males. Etiology can be attributed to primary psychiatric illness, substance abuse, neurological, or other medical conditions [2]. Symptoms can include delusions, hallucinations (most commonly auditory), disorganized thought and behavior, negative symptoms, and catatonia [3]. Long-term treatment of psychosis depends on the underlying cause and can include antipsychotics and mood stabilizers. These medications can have varying anticholinergic side effects such as constipation, dry mouth, blurred vision, urinary retention, and cognitive impairment. Constipation occurs through blockage of cholinergic receptors and thus smooth muscle contraction [4]. There is an association between increased anticholinergic burden and constipation, which has been shown to impair the quality of life in healthy patients and increase the risk of hospitalization in older patients [5]. Constipation can contribute to rectal prolapse, though rectal prolapse is rare in patients under age 50. Straining, and thus increased intra-abdominal pressure, is the likely mechanism of rectal prolapse when a patient is constipated. However, congenital defects in the musculature or connective tissues, perineal injury, pregnancy, and anatomic variations must also be considered as increasing the risk for rectal prolapse [6]. Although the exact relationship has yet to be distinguished, in patients with rectal prolapse, up to 50% of them have chronic psychiatric disorders, although none were on antipsychotic medications [7]. 

The aim of this report is to demonstrate a case of a female adolescent with psychosis and the complication of rectal prolapse and possible treatment.

## Case presentation

Our patient is a 17-year-old female who was admitted a second time to the adolescent inpatient behavioral unit for danger to self and grave disability due to psychosis. She presented with paranoia, delusions, and auditory/visual hallucinations. She had a previous stay in 2018 and had trials of outpatient therapy and medication management. However, she had not shown up to her most recent outpatient appointments. Her medications prescribed were 50 mg sertraline and 1 mg risperidone but adherence is unclear. Patient has a family history of depression and possible bipolar disorder. She lives at home with her mother, is estranged from her father, and attends high school. 

Prior to admission, the patient had been sleeping only 15 to 20 minutes a night for the past five nights. She believed she was possessed by demons and tried to eradicate the demons by climbing inside a heated oven. Also, the patient attacked her mother two days prior to admission, believing her mother had put glass in her food and water. The patient had not been eating and had been losing weight. She had multiple ED visits with the most recent visit five days prior to admission. Other medical causes had been ruled out during her ED visits. 

Since admission, the patient had been disoriented and paranoid. She would not accept offered food and medications. At the adolescent inpatient behavioral unit, patients are not forced to take medication. The patient believed the medications would turn her into a boy. She also refused to attend to her activities of daily living and was noted to have a disheveled, malodorous appearance. During the initial admission interview, the patient was not alert and oriented to place, time, or situation. She repeatedly stated, “God is here”. A thorough neurological exam did not show any Parkinsonian movements, choreoathetosis, ataxia, or cranial nerve palsies to suggest encephalitis or other neurologic pathology.

Although she began to cooperate with taking risperidone, she continued to have paranoid delusions. She continued to believe that there was a demon inside of her that she had to remove. She went to the bathroom often to push the demon out of her, eventually resulting in rectal prolapse. The patient was taken to the hospital on two different occasions due to the severity of prolapse, pain, and inability to stop the patient from pulling on the prolapse. However, during the emergency room visit, she was able to be redirected on how to push the prolapse in, did not report pain, and did not exhibit severe psychosis. There was no further treatment for the prolapse and the patient was sent back to the unit where she again displayed discomfort with the prolapse.

Despite one-to-one staffing and limits on the number of bathroom visits, there was continued concern that the patient was still attempting to "exorcize" the demon inside her body via the removal of rectal prolapse. The patient was started on 2 mg risperidone twice daily and 5 mg olanzapine as needed every four hours for severe agitation or psychosis. Her medication continued to be adjusted, then maintained throughout her stay. Overall, her treatment plan during the stay included one-to-one supervision, bathroom restrictions, bathroom supervision, bowel log completed by staff, and antipsychotic medications, which the patient refused on several occasions. Due to the patient’s level of psychiatric and medical needs, the patient was discharged on day eight of her stay, with the pursuit of alternative placement. 

After discharge from the inpatient unit, the patient presented to the ED with complaints of 13 events of intermittent seizure-like activity at home and altered mental status for two weeks. She also has been evaluated at Seattle Children’s hospital where she had two more instances of rectal prolapse. Physical exam and abdominal X-ray were negative. Complaints were most consistent with constipation. The patient was discharged with polyethylene glycol as needed for constipation and continued 2.5 mg am and 5mg pm of olanzapine. The subsequent outpatient follow-up did not report the patient having episodes of rectal prolapse with continued adherence to olanzapine but she continued to have symptoms of depression, auditory and visual hallucinations, decreased appetite, and suicidal ideation.

## Discussion

We report on a 17-year-old female with psychosis who has complications of rectal prolapse. The patient was having delusions about a demon inside her and this caused her to frequently use the toilet to get rid of the demon. She eventually developed a rectal prolapse, even though rectal prolapses are rare before the age of 50. Following the prolapse, the patient was having more paranoia due to being able to see the prolapse and associating it with the demon so she tried to pull the prolapse out. Now this posits the question of how the treatment should proceed. If the rectal prolapse was surgically treated, would the patient’s paranoia decrease since she would no longer see the "evil" coming out of her? Did the anticholinergic side effects of constipation in the antipsychotic medications exacerbate her delusions of having a demon inside her? If the constipation was prophylactically treated, would this have prevented the prolapse from happening in the first place? Could the complication of rectal prolapse have been avoided if she was forced to take the medication? And finally, though the patient initially had a negative neurologic workup, was it possible that she was having a rare presentation of anti-NMDA receptor encephalitis?

The first question can possibly be answered through a retrospective cohort study. Patients under the age of 50 with rectal prolapse underwent surgical treatment and it was concluded that there was a higher risk of recurrence and constipation post-surgery in patients with severe psychiatric diseases on long-term medication [8]. As for the second question, it is plausible that if the patient was able to forcibly receive antipsychotic medication early on, the prolapse may have never developed. If her hallucinations were managed, she may have not strained to push them out, leading to rectal prolapse. Medical treatment may be the more ideal option for this patient since there are some initial studies suggesting worse outcomes in older children and adolescent patients who undergo surgical repair for rectal prolapse [9-11]. Higher incidences of recurrence were also associated with increased age in pediatric patients [9]. In our case, the facility she was placed in could not force medication and therefore, it had to be on the patient’s own accord to take the medication. With the patient’s young age, a poor functional outcome from a surgical repair would not be ideal and potentially harmful to an adolescent with a psychiatric disorder. Instead, management was focused on antipsychotic medication, one-to-one care for the patient to prevent physical harm, and deferral to a higher acuity facility for rectal prolapse where antipsychotic and bowel regimen medications were administered. Finally, it is possible that initiating early or prophylactic treatment for constipation could have prevented serious side effects [8, 12]. In our case, when our patient was first started on risperidone, prophylactic treatment of constipation could have helped subside some of the patient’s symptoms and hallucinations involving her GI system. Due to incomplete records, we were unable to determine whether the patient’s previous psychotic delusions of the demon inside her occurred prior to initiation of her medication treatment or after and were unable to determine the patient's fiber and fluid intake at home. Moreover, early detection of constipation is difficult because patients with psychotic disorders often have higher pain thresholds and/or lower levels of self-awareness [13]. For this patient, the resolution of psychosis most likely factored into her recurrence of rectal prolapse, however, it is also possible that the addition of polyethylene glycol to her medication could have helped as well. As for anti-NMDA receptor encephalitis, the patient had pathognomonic neuropsychiatric symptoms, including disturbances of consciousness, paranoia, delusions, and catatonia [14]. She also had possible seizures that can also be caused by anti-NDMA receptor encephalitis. However, many presentations of this encephalitis are associated with recent vaccinations, and our patient had no recent immunization history or sudden onset of these symptoms. Additionally, further workup, including EEG, was inconclusive. However, it would be important to follow up with cerebrospinal fluid analysis and possibly MRI if our patient presented with these acute symptoms again in the future. 

## Conclusions

Psychosis is a complex issue that can cause a variety of complications and requires a multifactorial approach to resolution. For this patient there was a perfect storm of problems where psychosis made her want to push the demon out causing increased intra-abdominal pressure, causing prolapse. At the same time, she was given antipsychotic medications that had a side effect of constipation. She continually tried to strain on the toilet until she had a rectal prolapse which inadvertently worsened her psychosis and caused her to want to push out the demon more. Although this situation was complex, antipsychotic medication adherence and an additional bowel regimen helped prevent recurrent rectal prolapse and improved the psychotic symptoms. Constipation is a common side-effect of antipsychotic medications. Regardless of age, in patients especially with psychosis, symptoms of constipation should be monitored closely and treated with preventative measures such as diet, hydration, and physical activity. In the case of rectal prolapse, conservative treatment is best for an actively adolescent psychotic patient as surgical options seem to show high rates of recurrence.
